# Intercellular and inter-organ crosstalk in browning of white adipose tissue: molecular mechanism and therapeutic complications

**DOI:** 10.1093/jmcb/mjab038

**Published:** 2021-06-29

**Authors:** Lai Yee Cheong, Aimin Xu

**Affiliations:** 1 The State Key Laboratory of Pharmaceutical Biotechnology, The University of Hong Kong, Hong Kong, China; 2 Department of Medicine, The University of Hong Kong, Hong Kong, China; 3 Department of Pharmacology and Pharmacy, The University of Hong Kong, Hong Kong, China

**Keywords:** adipose tissue browning, circulating factors, energy homeostasis, adipose biology, obesity

## Abstract

Adipose tissue (AT) is highly plastic and heterogeneous in response to environmental and nutritional changes. The development of heat-dissipating beige adipocytes in white AT (WAT) through a process known as browning (or beiging) has garnered much attention as a promising therapeutic strategy for obesity and its related metabolic complications. This is due to its inducibility in response to thermogenic stimulation and its association with improved metabolic health. WAT consists of adipocytes, nerves, vascular endothelial cells, various types of immune cells, adipocyte progenitor cells, and fibroblasts. These cells contribute to the formation of beige adipocytes through the release of protein factors that significantly influence browning capacity. In addition, inter-organ crosstalk is also important for beige adipocyte biogenesis. Here, we summarize recent findings on fat depot-specific differences, secretory factors participating in intercellular and inter-organ communications that regulate the recruitment of thermogenic beige adipocytes, as well as challenges in targeting beige adipocytes as a potential anti-obese therapy.

## Introduction

Obesity is a global health concern due to its association with noncommunicable diseases such as type 2 diabetes, cardiovascular diseases, and cancers. Despite there being a range of interventions and intensive research efforts in the field, the prevalence of obesity has reached epidemic proportions globally. Owing to the continuous increase in the number of individuals adopting a sedentary lifestyle in both developed and developing countries, over 107 million children and 603 million adults are affected by obesity worldwide (Collaborators, 2017).

Obesity occurs when energy intake chronically exceeds energy expenditure. Excess nutrients in the body get stored in AT in the form of triglycerides. AT takes up ∼20% of the total body weight in healthy adults as opposed to obese individuals, which can exceed 40% in order to facilitate the storage of excess energy (Ikeda et al., 2018). As a metabolically active organ, AT can undergo structural and cellular remodeling when exposed to different environmental cues, such as dietary and temperature changes. Thus, most of the currently available anti-obesity medications act through suppressing appetite or the inhibition of intestinal lipid absorption in order to limit energy intake. However, such medications are always associated with adverse side effects, such as steatorrhea, depression, and heart diseases (Cheung et al., 2013). In recent decades, a growing body of evidence obtained from both animal and clinical studies suggests that the activation of brown adipose tissue (BAT)-mediated adaptive thermogenesis or non-shivering thermogenesis is a plausible strategy to counteract body weight gain and maintain glucose homeostasis (Lowell et al., 1993; Rossmeisl et al., 2002; Stanford et al., 2013). In 2009, the presence of functional BAT in adult humans was first reported by measuring ^18^F-fluorodeoxyglucose (^18^F-FDG) uptake using positron-emission tomography and computed tomography (PET/CT) imaging (Cypess et al., 2009), which has long been believed to only exist in infants. Subsequent studies demonstrated that the amount and activity of BAT were inversely correlated with body mass index (BMI), aging, and metabolic risk (Cypess et al., 2009; Saito et al., 2009; Yoneshiro et al., 2013), indicating the importance of BAT in regulating energy metabolism and homeostasis in humans.

Although results from early studies suggest the presence of BAT in adult humans, recent transcriptional analyses of human BAT biopsies have revealed a distinct type of adipocytes that express a molecular signature specific to another cell type, known as inducible beige/brown-like adipocytes (Sharp et al., 2012). Other studies have reported the presence of beige adipocytes in the supraclavicular region of adult humans who do not possess detectable pre-existing BAT before cold exposure (van der Lans et al., 2013; Yoneshiro et al., 2013). In addition, cold exposure seems to promote a wider distribution of glucose uptake in AT than previously identified BAT regions in adult humans (Leitner et al., 2017). Furthermore, the importance of beige adipocytes in energy homeostasis and thermoregulation has been demonstrated with the finding that the genetically modified mice that exhibit dysfunctional classical BAT but show compensatory beiging/browning in subcutaneous WAT to maintain whole-body temperature are protected against high-fat diet (HFD)-induced obesity (Schulz et al., 2013). Therefore, the formation of inducible beige adipocytes by converting WAT into thermogenic active beige fat depots may be a promising therapeutic approach given the excess amount of WAT in obese individuals. However, the depot-specific browning capacity of WAT may become a hurdle and limit its therapeutic value. For instance, visceral fat is less susceptible than subcutaneous fat to the browning process. Thus, it is crucial to understand the switching capacity of WAT from energy storage to energy dissipation. In this review, we aim to discuss the recent research progress on beige adipocytes, factors that influence their development, and unresolved questions in this field.

## Basic features of beige adipocytes

Previously, AT was only thought to provide energy storage and mechanical protection for multiple body sites. In the last decade, researchers have discovered an additional role of WAT as a thermogenic effector organ. To this end, at least in the mouse model, three types of functionally distinct adipocytes have been identified and well characterized, including white, brown, and beige. Unilocular white adipocytes are primarily involved in fat storage and utilization, whereas multilocular brown adipocytes dissipate chemical energy as heat and have a high basal level of thermogenic factor known as mitochondrial uncoupling protein 1 (UCP1). Beige adipocytes have been found to exist in mouse inguinal subcutaneous fat and human supraclavicular regions. They are inducible UCP1^+^ cells that appear to intersperse in WAT but not *bona fide* brown adipocytes (Sharp et al., 2012; Wu et al., 2012; Shinoda et al., 2015).

### Anatomical location

**Figure 1:**
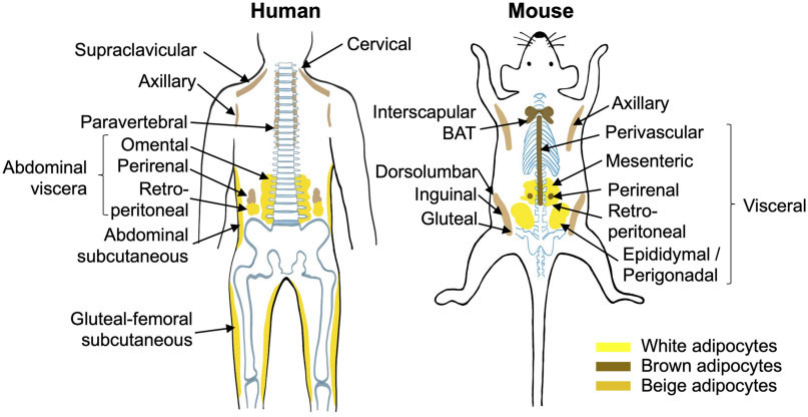
Distribution of fat tissues in the human and mouse. In human, adipose depots are divided into upper body subcutaneous (cervical, supraclavicular, axillary, paravertebral, and abdominal subcutaneous), abdominal viscera, and lower body subcutaneous (gluteal-femoral subcutaneous). In mouse, adipose depots are generally distributed into anterior subcutaneous (axillary and BAT), visceral, and posterior subcutaneous (dorsolumbar, inguinal, and gluteal).

Beige adipocytes contain many small lipid droplets and dense mitochondria, but have a comparatively low basal expression of UCP1. Nevertheless, UCP1 expression in beige adipocytes can be induced to a level comparable to that in BAT after appropriate stimulants such as cold exposure, exercise, and beta 3-adrenergic receptor (β3-AR) or peroxisome proliferator-activated receptor gamma (PPARγ) agonists (Boström et al., 2012; van der Lans et al., 2013; Cypess et al., 2015; Merlin et al., 2018). Unlike BAT, which have defined anatomical locations (e.g. intrascapular and perirenal regions) in mice, beige adipocytes are scattered within WAT. In rodents, beige adipocytes are mainly found in the posterior inguinal WAT and become more prominent upon prolonged cold exposure or in response to other stimulants. In humans, beige/brown adipocytes have been observed in the upper body trunk such as supraclavicular, paravertebral, cervical, axillary, and perivascular regions (Cypess et al., 2009; Shinoda et al., 2015; Ikeda et al., 2018). In addition, since beige adipocyte biogenesis utilizes glucose and fatty acids, additional active beige depots have been observed in adult humans through CT scans using the iodine-123-β-methyl-ρ-iodophenyl-pentadecanoic acid (^123^I-BMIPP) fatty acid uptake approach, including anterior subcutaneous and suprascapular regions (Zhang et al., 2018). Although active UCP1^+^ adipocytes in humans resemble both brown and beige adipocytes, mounting evidence suggests that they possess molecular features similar to the latter phenotype (Wu et al., 2012; Jespersen et al., 2013; Lee et al., 2014b; Shinoda et al., 2015). Yet, it is still difficult to distinguish between brown and beige adipocytes in adult humans using current available approaches (e.g. PET/CT scanning) due to the highly heterogenous population of UCP1^+^ adipocytes, variations of molecular characteristics across different fat depots, and obvious differences in anatomical location and distribution of adipose depots between mice and humans (Figure 1).

### Developmental origins

The thermogenic beige adipocytes have distinct cellular origins from brown and white adipocytes. It has been long proposed that beige adipocytes emerge from pre-existing mature white adipocytes through a transdifferentiation process. Initially, this concept was supported by studies showing an insignificant proliferation rate of adipocytes during the browning process (Himms-Hagen et al., 2000; Barbatelli et al., 2010; Vitali et al., 2012). Subsequently, a genetic lineage-tracing study demonstrated that cold-induced UCP1^+^ multilocular beige adipocytes in inguinal WAT switched to the unilocular white adipocyte phenotype during warm exposure. When these adipocytes were subjected to cold exposure again, a conversion to the beige phenotype was observed, indicating an instant response of hypoactive or dormant beige adipocytes in WAT during environmental changes (Rosenwald et al., 2013). Furthermore, independent studies have revealed molecular mechanisms that regulate the transition of beige-to-white phenotype after withdrawing stimuli, which have been shown to involve autophagy-mediated mitochondrial clearance (Altshuler-Keylin et al., 2016) and the transcription factor zinc-finger protein, ZFP423 (Shao et al., 2016; Roh et al., 2018).

On the other hand, a growing number of studies suggest that beige adipocytes originate from *de novo* differentiation of precursor cells (Sanchez-Gurmaches et al., 2012; Wang et al., 2013; Sanchez-Gurmaches and Guertin, 2014b). Scherer and colleagues provided the first evidence for the existence of such precursor cells by marking and tracking the fate of mature adipocytes during cold exposure or treatment with the β3-AR agonist through a pulse‒chase lineage-tracing mouse model approach (Wang et al., 2013). Subsequent studies also support the notion that beige adipocytes arise from resident precursor cells. For instance, myogenic factor 5-negative (Myf5^–^) or Myf5^+^ lineage precursor cells commit and differentiate into beige adipocytes (Sanchez-Gurmaches et al., 2012; Sanchez-Gurmaches and Guertin, 2014b). In addition, subsets of beige adipocytes exhibit a smooth muscle-like gene molecular signature (e.g. MYH11, ACTA2, PDGFRα, and PDGFRβ) in inguinal, axillary, and epididymal WAT after acute or chronic cold adaptation, indicating that vascular smooth muscle or mural cells are attributable to the beige adipocyte pool (Lee et al., 2012; Long et al., 2014; Vishvanath et al., 2016). Furthermore, capillary sprouts from explanted human subcutaneous WAT were able to differentiate into beige adipocytes, and subsequent subcutaneous transplantation of these *ex vivo* differentiated beige adipocytes into dietary obese mice improved systemic glucose homeostasis (Min et al., 2016). However, the precise origin of beige adipocytes remains unclear and needs further investigation.

Despite numerous studies aiming to determine the predominant developmental mechanism of beige adipocytes, it remains inconclusive. This is due to differences in mouse strains (Collins et al., 1997; Xue et al., 2007), types of browning stimuli (Jiang et al., 2017b), and lineage-tracing systems (Ye et al., 2015; Zhao et al., 2020) used among studies, which may impact the outcomes and interpretations of results. Recently, Gupta and colleagues shed light on this issue by showing that different animal-housing temperatures before treatment with β3-AR agonist (CL316,243) or cold exposure led to differing modes of beige cell recruitment (Shao et al., 2019). Both transdifferentiation and *de novo* differentiation were apparent in inguinal WAT of AdipoChaser mice after direct animal transition from room temperature (∼22°C) to a cold environment (∼6°C). Strikingly, over 80% of beige adipocytes emerged from *de novo* differentiation after mice were exposed to thermoneutral condition (∼30°C) for 4 weeks prior to cold exposure. However, animal-housing conditions did not influence beige adipocyte biogenesis induced by CL316,243, which favored the direct conversion of white-to-beige adipocytes. The authors suggested that precursor-derived beige adipocytes dominate upon the first cold exposure and rapidly respond by interconverting between ‘unilocular quiescent beige’ and ‘multilocular active beige’ states following further environmental changes. Together, these findings emphasize that the inducibility of beige adipocytes is highly dependent on the type of thermogenic stimuli and temperature of the exposure before cold adaptation.

## Cellular heterogeneity of AT

Owing to technological advances such as *in vivo* lineage-tracing models and single-cell RNA sequencing (scRNA-seq), it has been well established that AT is a highly heterogenous organ that may contribute to different browning capacities among fat depots as well as within the same depot.

### Inter-depot difference

It is widely accepted that subcutaneous WAT, but not visceral WAT, can undergo extensive browning in response to cold exposure in mice. These differences in browning capacity of discrete adipose sites have triggered considerable interest in uncovering the fundamental properties of WAT, especially visceral/epididymal and subcutaneous WAT. WAT is a heterogenous endocrine organ consisting of adipocytes and numerous cell types such as nerve fibers, immune cells, stromal cells, and blood vessels (Thiagarajan and Reizes, 2016). The heterogeneous cell types in WAT may explain in part different degrees of beiging capacity among and within different fat depots. First, different capacities of sympathetic nervous system (SNS) innervation in different adipose depots may lead to significant differences in the browning ability of WAT. This has been clearly demonstrated by several recent publications showing that dense SNS directly innervates subcutaneous WAT, but not epididymal WAT, following cold exposure, as indicated by immunolabelling of tyrosine hydroxylase (TH, marker of SNS neurons) and UCP1 using whole-mount tissue clearing (AdipoClear) with a light-sheet microscopy (Jiang et al., 2017a; Chi et al., 2018). Likewise, sympathetic denervation of subcutaneous WAT impairs the browning process (Contreras et al., 2014). Second, the subcutaneous depot displays higher vascular density than visceral fat in response to cold exposure or adrenergic receptor activation (Xue et al., 2009). A previous study has demonstrated blood vessel walls to be a source of beige adipocyte progenitors, which are able to differentiate into mature beige cells in conjunction with pro-angiogenic factor-stimulated capillary network expansion in an *ex vivo* system (Min et al., 2016). These findings are further supported by the discovery of platelet-derived growth factor receptor (PDGF-CC)-secreting endothelial cells during angiogenesis in WAT. PDGF-CC regulates the differentiation of platelet-derived growth factor receptor alpha-positive (PDGFRα^+^) bi-potential progenitor cells into beige adipocytes in subcutaneous WAT (Seki et al., 2016). Third, adipocytes do not share a common developmental origin. Lineage tracing in vivo has revealed that the Wilms tumor (Wt1) gene, an important regulator of mesenchymal progenitor cell activity, is expressed in different depots of visceral fat but not in subcutaneous fat and BAT in mice (Chau et al., 2014). Moreover, >95% of brown adipocytes arise from Myf5^+^ lineage precursors, while most white adipocytes originate from Myf5^–^ precursors in WAT (Sanchez-Gurmaches and Guertin, 2014a, b). Consistently, large-scale transcriptomic analysis of human WAT also revealed inter-WAT depot differences (Tchkonia et al., 2007; Vijay et al., 2020). In addition, distinct cellular compositions in various fat depots have been identified in mice and human adipose samples (Schwalie et al., 2018). For instance, significant differences in immune cell populations have been observed between human visceral and subcutaneous fat. In particular, T/natural killer (NK) cells, monocytes, and macrophages were shown to be remarkably higher in subcutaneous WAT than in visceral WAT (Vijay et al., 2020). Finally, the recruitment mechanism of beige adipocytes may vary in different WAT depots. A recent study demonstrated that beige adipocytes are transdifferentiated from resident white adipocytes in subcutaneous WAT (Rosenwald et al., 2013), whilst the majority of beige adipocytes derive from *de novo* biogenesis from bipotential progenitors in epididymal WAT (Lee et al., 2012). However, to date, no definitive conclusion has been reached on this matter due to the complex factors influencing the development of beige adipocytes.

### Intra-depot variation

Recent technology advances in high-throughput sequencing have revealed distinct types of adipocytes and various cellular compositions within an adipose depot. For instance, 14 clusters of adipocytes within a subcutaneous fat were found to express different gene markers involved in different functional processes including thermogenesis (Rajbhandari et al., 2019), highlighting heterogenous distribution within a fat depot. In addition to transcriptomic profiling, cold-induced beige adipocyte biogenesis occurs in the core region of subcutaneous WAT close to the lymph node and gradually shifts to other regions after chronic cold exposure, as visualized by high-resolution images (Barreau et al., 2016; Chi et al., 2018). However, to date, no follow-up studies have been reported to find out the reasons for this regional variation in the browning process, which happens in rodents. Further investigation on this interesting observation may yield additional valuable information on the browning capacity of WAT.

## Microenvironment in WAT for the development of beige adipocytes

In adult humans, BAT rarely exists as it does in mice, possibly due to the long-term exposure to a thermoneutral environment. The thermogenic cells in adult humans are believed to be inducible beige adipocytes as they share similar molecular signatures with mouse beige adipocytes. It is also well-established that active beige adipocytes can be recruited in fat depots of both mice and humans following the appropriate thermogenic signals/stimuli. Thus, the microenvironment in the fat depots is an important determinant in their browning capacity.

**Figure 2:**
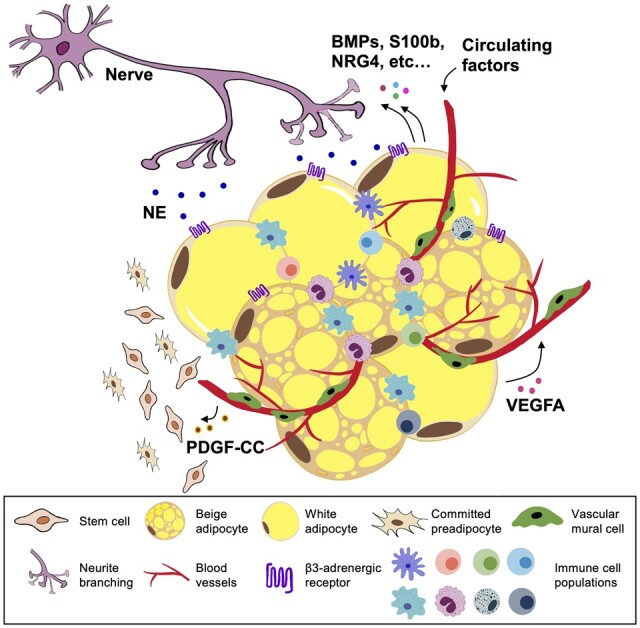
Heterogeneity, cell‒cell communications, and microenvironment within WAT in the development of beige adipocytes. The crosstalk amongst different cell populations is essential for the formation and activation of beige adipocytes. In response to thermogenic stimuli (such as cold exposure), sympathetic innervation releases NE to promote β3-AR-expressing adipocytes for lipolysis and thermogenic programs. On the other hand, adipocytes produce neurotrophic factors to stimulate neurite branching. Similarly, adipocytes also secrete VEGFA to promote angiogenesis for increased supply of nutrition and oxygen during thermogenesis, as well as for creating a niche favorable for adipocyte progenitors. Endothelial cells release PDGF-CC to act on PDGFRα^+^ progenitors for adipogenesis of beige adipocytes. Furthermore, infiltration and activation of type 2 immunity signaling (e.g. ILC2s, eosinophils, and M2 macrophages) also drive the beige fat biogenesis.

Beige adipogenesis is generally a two-step process: commitment and differentiation. Committed beige preadipocytes arise from multipotent mesenchymal stem cells (MSCs) and terminally differentiate into mature adipocytes in response to certain transcription factors. Apart from gene control mechanisms, the local microenvironment in WAT plays a vital role in the development of beige adipocytes. The recruitment, activation, and maintenance of beige adipocytes are regulated by a tight coordination and synchronization among various cellular components in WAT, for example, endothelial, nerve, immune, and adipocyte cells (Figure 2).

### AT vascularization

AT is highly vascularized during the development of beige adipocytes. In addition to providing a constant supply of oxygen and nutrients, substantial evidence suggests that the vascular cell walls in AT are the niche of adipocyte progenitors. Notably, the identification of Zfp423^+^ committed preadipocytes that exist in mice and humans are shown to originate from mural cells lining the adipose capillary endothelium, which can undergo bipotential differentiation into white or brown adipocytes (Gupta et al., 2012; Tran et al., 2012). Furthermore, it has been proven that beige adipocytes derived from capillary networks in human subcutaneous WAT express thermogenic-related genes. Transplantation of these *in vitro* differentiated beige adipocytes further enhances systemic glucose tolerance in dietary obese mice (Min et al., 2016). Apart from serving as a reservoir of stem cells, angiogenic endothelial cells also produce PDGF-CC that acts on PDGFRα^+^ progenitors to promote WAT browning upon cold challenge or stimulation with β3-AR agonists (Seki et al., 2016). In addition, cold-induced angiogenesis occurs in subcutaneous WAT and this is accompanied by increased mRNA expression of vascular endothelial growth factor A (VEGFA), protein expression of UCP1, and beige appearance with multilocular lipid structure (Xue et al., 2009). Adipocyte-specific overexpression of VEGFA using doxycycline-inducible mouse model robustly enhanced beiging of subcutaneous WAT (Park et al., 2017). Consistently, VEGFA or vascular endothelial growth factor receptor 2 (VEGFR2) blockade abolishes the browning effect and impairs adaptive thermogenesis (Xue et al., 2009). In contrast, endothelial cell-specific ablation or pharmacological inhibition of VEGFR1 resulted in robust WAT angiogenesis and browning effect. The enhanced angiogenesis and browning effects in AT of VEGFR1-deficient mice could be explained by the role of VEGFR1 as a decoy receptor of VEGFA (Cao, 2009), and thus it may indirectly inhibit the proangiogenic activity of VEGFA. It is also possible that the ablation of VEGFR1 could have switched VEGFA to VEGFR2 signaling and enhanced the effect of VEGFR2-mediated angiogenesis, thereby increasing the biogenesis of beige adipocytes (Seki et al., 2018). Therefore, these findings support the notion that the growth of capillaries and proangiogenic factors play pivotal roles in modulating the recruitment of beige adipocytes in WAT.

### AT innervation

Like BAT, SNS is a potent activator in the formation of beige adipocytes. During cold exposure, the amount of sympathetic nerve endings increases in subcutaneous WAT, as evidenced by a higher expression level of TH, which leads to the release of catecholamines, especially norepinephrine (NE), to the surrounding WAT (Morrison, 2016). Subsequently, NE acts on the β3-AR to activate the cyclic adenosine 3′,5′-monophosphate/protein kinase A (cAMP/PKA) signaling cascade pathway, which in turn drives lipolysis and thermogenic processes (Fedorenko et al., 2012; Zeng et al., 2015). Surgical or chemical denervation of subcutaneous WAT in mice using neurotoxin 6-hydroxydopamine caused a dramatic reduction in UCP1 expression and NE levels in the subcutaneous fat of mice (Contreras et al., 2014; Ding et al., 2016). One early study showed that genetic ablation of β3-AR in mice led to an impairment in WAT browning (Jimenez et al., 2003). In contrast, two other studies found that β3-AR knockout (KO) mice or mice lacking all β-ARs exhibit intact formation of beige cells in subcutaneous WAT upon cold acclimation (de Jong et al., 2017; Chen et al., 2019). The different outcomes of cold-induced browning in β3-AR KO mice could be possibly attributed to the different genetic backgrounds in mouse models. The mice used by Jimenez et al. (2003) were on C57BL/6J background whereas the mice generated by de Jong et al. (2017) were on FVB/N background. It has been reported that different mouse strains exhibit significant differences in several metabolic parameters including cold-induced changes in BAT and WAT (Ferrannini et al., 2016). In addition, different cold exposure regime can also lead to different outcomes (Cui et al., 2016). In the mouse model lacking all the three forms of β-ARs, a compensatory pathway of beige fat biogenesis has been identified, which enables the mice to survive during gradual cold acclimation (Chen et al., 2019). Recently, several studies have demonstrated an interaction between SNS and adipocytes by identifying adipocyte-derived neurotrophic factors such as calsyntenin-3β/S100b (Zeng et al., 2019), bone morphogenetic proteins (BMPs) (Schulz et al., 2013; Pellegrinelli et al., 2018), and neuregulin-4 (NRG4) (Rosell et al., 2014) that promote the growth and branching of sympathetic nerves during cold-induced browning in subcutaneous fat. In addition, a mouse model with adipocyte-specific deletion of PR-domain containing 16 (Prdm16), a major transcriptional regulator of beige adipogenesis, displayed an impaired browning phenotype and a drastic reduction in sympathetic neurite density (Chi et al., 2018), suggesting that beige adipocytes interact with neurons via secreted factors to activate and maintain the function of thermogenic cells. Taken together, these findings support the notion that the dialogue between SNS and adipocytes is essential to modulate beige biogenesis and thermogenic activation in WAT.

### Immune cells in WAT

**Figure 3:**
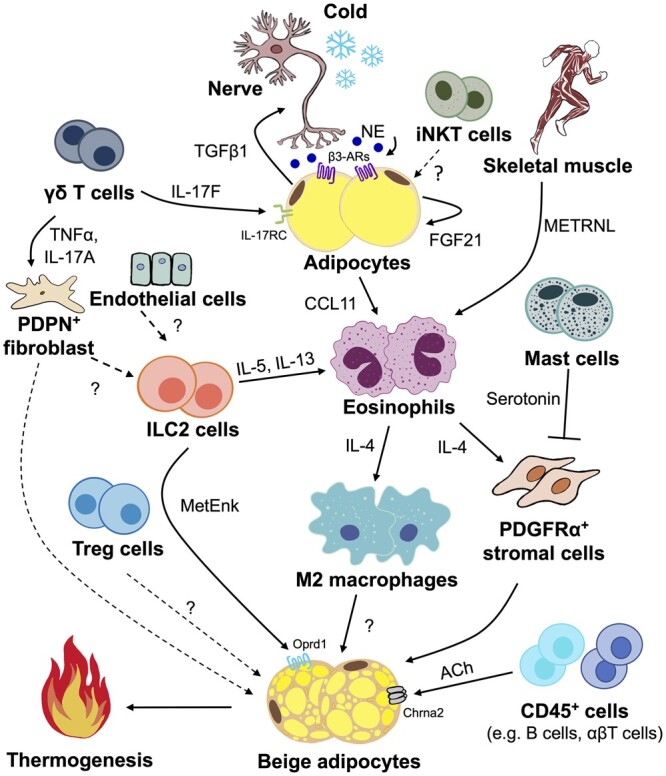
Immune cells and their secreted cytokines in the biogenesis of thermogenic beige adipocytes in WAT. In response to thermogenic stimuli such as cold exposure and high-intensity exercise, type 2 immune responses characterized by ILC2s, eosinophils, and alternatively activated (M2) macrophages are activated to secrete type 2 cytokines and other factors for induction of the beiging program. Furthermore, different T cell subsets (γδ T cells, iNKT cells, and Treg cells), mast cells, and CD45^+^ cells also secrete respective factors to modulate biogenesis of beige adipocytes. Question mark (?) represents unknown factors. TNFα, tumor necrosis factor alpha.

Immune cells present in the stromal vascular fraction (SVF) of WAT have long been known to be involved in the homeostasis of WAT. Similar to other organs, WAT is subjected to immune surveillance in response to environmental cues. Obesity provokes type 1 immune response and gradually leads to the development of low-grade, chronic inflammation, whereas in healthy lean individuals, type 2 immunity is predominantly responsible for restraining inflammation and maintaining metabolic homeostasis. Transcriptomics profiling and pathway enrichment analysis of progenitor-derived beige adipocytes from human WAT explant revealed the expression of cytokines suggestive of type 2 immunity (e.g. IL-11 and CXCL8), which may be important for the intercellular crosstalk between immune and mesenchymal progenitor cells in WAT (Min et al., 2019). Thus, immune cells play important roles in the regulation of beige adipogenesis (Figure 3).

#### Macrophages

Adipose tissue macrophages (ATMs) are the main leukocyte population in WAT (Nishimura et al., 2009), which are typically classified as ‘classically activated’ M1 or ‘alternatively activated’ M2. The M2 macrophages predominantly possess anti-inflammatory phenotype in lean subjects whereas in obese individuals, macrophages preferentially polarize into pro-inflammatory M1 macrophages in WAT (Lumeng et al., 2007). A recent study has shown the communication between classical BAT and WAT during cold acclimation, whereby CXCL14 secreted by brown adipocytes targets peripheral WAT depots to promote the recruitment of M2 macrophages and subsequently lead to WAT browning (Cereijo et al., 2018). Furthermore, the increased abundance of M2 macrophages in AT during cold-induced beige adipogenesis has been consistently observed (Hui et al., 2015; Huang et al., 2017; Qian et al., 2018). However, the mechanism whereby M2 macrophages regulate the formation of beige adipocytes is still controversial. An early study demonstrated that IL-4/IL-13 signaling induces the polarization of M2 macrophages to release catecholamines and ultimately favors the browning of WAT during acclimation to cold (Nguyen et al., 2011). In contrast, another recent study argued that M2 macrophages do not express TH, a rate-limiting enzyme required for NE production, and thus excluding M2 macrophages as a potential source of NE during cold-induced browning of WAT (Fischer et al., 2017). Instead, this study proposed that M2 macrophages may regulate cold-induced browning of WAT by uptaking and releasing NE (Fischer et al., 2017). Therefore, further investigations are needed to find out the precise mechanism(s) whereby M2 macrophages promote the formation of beige adipocytes.

#### Eosinophils

The IL-4 signaling pathway is well known to be a key player in the polarization and proliferation of tissue-resident M2 macrophages (Rückerl et al., 2012; Jenkins et al., 2013). The major IL-4-secreting cells has been reported to be eosinophils, which constitute ∼4%‒5% of the SVF of lean subcutaneous WAT and appear to modulate systemic glucose (Wu et al., 2011). Two independent studies discovered the importance of eosinophils in the recruitment and activation of beige adipocytes in WAT by using an eosinophil-deficient mouse model (ΔdblGATA) (Qiu et al., 2014; Rao et al., 2014). Cold exposure not only increases the number of eosinophils and expression of type 2 cytokines (e.g. IL-4 and IL-13), but also induces the infiltration of macrophages expressing the chemokine receptor CCR2 into subcutaneous WAT (Qiu et al., 2014). A circulating factor known as meteorin-like (METRNL), which is mainly secreted from skeletal muscle and AT, was found to stimulate the recruitment of eosinophils into subcutaneous WAT, which in turn leads to the polarization of M2 macrophages and eventually browning of WAT in response to exercise or cold acclimation (Rao et al., 2014). Moreover, it has been reported that cold-induced recruitment of eosinophils into WAT is mediated by adipocyte-secreted FGF21, which acts in an autocrine or paracrine manner to induce the production of C‒C motif chemokine 11 (CCL11), a key chemokine responsible for eosinophil recruitment (Huang et al., 2017). Apart from the activation of M2 macrophages, IL-4-producing eosinophils can directly act on IL-4Rα-expressing PDGFRα^+^ adipocyte progenitors that commit to the beige cell lineage (Lee et al., 2015a).

#### Group 2 innate lymphoid cells (ILC2s)

Two independent research groups discovered the involvement of ILC2s in regulating thermogenic activation of beige adipocytes (Brestoff et al., 2015; Lee et al., 2015a). ILC2s originate from common lymphoid progenitors and lack the conventional B or T cell receptors. In response to the cytokine IL-33 (Neill et al., 2010), ILC2s secrete IL-5 and IL-13 and coordinate with eosinophils to elicit type 2 immune response during lung and gastrointestinal nematode infections (Yasuda et al., 2012; Wojno et al., 2015). Notably, the number of ILC2s in WAT was reduced in both humans and mice with obesity (Brestoff et al., 2015). Administration of IL-33 increased the abundance of ILC2s and promoted the browning of WAT in mice (Brestoff et al., 2015; Lee et al., 2015a). *Vice versa*, IL-33-deficient mice exhibited a decreased abundance of ILC2s and were associated with the impairment of browning capacity in WAT (Brestoff et al., 2015). In addition to the secretion of type 2 cytokines, ILC2s also produce an opioid-like peptide called methionine‒enkephalin (MetEnk), which bypasses the type 2 cytokine signaling pathway and directly acts on adipocytes to promote the formation of functional beige adipocytes (Brestoff et al., 2015). Furthermore, it has been suggested that this pathway occurs selectively in subcutaneous WAT that expresses higher levels of the MetEnk receptor (known as opioid receptor δ1, Oprd1) compared to classical BAT (Brestoff et al., 2015). Although IL-33 is known to be produced by epithelial cells (Moussion et al., 2008; Pichery et al., 2012), podoplanin (PDPN) fibroblasts (Pichery et al., 2012), and cadherin-11 cells (Chang et al., 2017), the source of endogenous IL-33 during beige adipogenesis in WAT remains largely unknown.

#### T cells

Different T cell subsets are present in AT. An unconventional innate T cell subpopulation, type 1 or invariant natural killer T (iNKT) cell, is enriched specifically in human and murine AT and produces anti-inflammatory cytokines, for example, IL-10 and IL-4 (Lynch et al., 2012). The abundance of adipose iNKT cells is reduced in obesity but restored after weight loss in humans and mice (Lynch et al., 2012). It has been demonstrated that iNKT cells regulate the formation of thermogenic beige adipocytes in WAT partially through the iNKT‒FGF21 axis (Lynch et al., 2016). However, the exact mediator(s) secreted from iNKT cells that promote beige biogenesis have yet to be identified. Nevertheless, it is important to note that the lipid ligand α-galactosylceramide-mediated activation of iNKT cells enhances polarization of M2 macrophages via the IL-4/STAT6 signaling pathway, thus improving systemic glucose homeostasis in dietary obese mice (Ji et al., 2012). Another type of innate lymphocyte, γδ T cell, is also highly enriched in WAT of both mice and humans and negatively correlated with BMI (Costanzo et al., 2015; Kohlgruber et al., 2018). Because γδ T cells are the main source of IL-17A during cold acclimation, genetic deficiency of IL-17A altered cold-induced thermogenic responses in subcutaneous WAT by increasing the abundance of adipose PDPN^+^ stromal cells that can differentiate into beige adipocytes (Kohlgruber et al., 2018). A recent study further highlighted the role of γδ T cells in adaptive thermogenesis by discovering its function in promoting sympathetic innervation through IL-17F-driven transforming growth factor beta 1 (TGFβ1) signaling via the adipocyte-expressed IL-17 receptor C (IL-17RC) (Hu et al., 2020). In addition, the expansion of differentiated regulatory T (Treg) cells from CD4^+^ T cells also regulates thermogenesis in subcutaneous WAT through the STAT6/PTEN signaling axis after cold or β3-AR stimulation, as evidenced by increased lipolysis, β-oxidation, and thermogenic gene expression (Kälin et al., 2017). In contrast, CD4^+^Foxp^+^ Treg cells have been shown to produce a significant amount of IL-10 to suppress beiging of WAT (Beppu et al., 2020). A follow-up study also demonstrated that the ablation of IL-10 or adipose-specific depletion of IL-10Rα in mice led to greater induction of browning in subcutaneous WAT than their wild-type littermates (Rajbhandari et al., 2018). Further studies are necessary to investigate the regulator(s) of Treg cells in modulating beige biogenesis as IL-10 production can be produced by many types of immune cells in the adaptive immune system (Saraiva and O’Garra, 2010).

#### Mast cells (MCs)

Although MCs are known for their role in allergic reactions, their abundance in WAT has been found to be increased in obese mice and humans (Liu et al., 2009). In addition, the number of MCs was elevated in thigh subcutaneous fat of human subjects during the winter season (Finlin et al., 2017). By contrast, a scRNA-seq revealed a decreased abundance of MCs in subcutaneous fat of mice after treatment with the β3-AR agonist, CL316,243 (Rajbhandari et al., 2019). Intriguingly, functional inactivation or genetic deficiency of MCs promoted adipocyte browning and increased systemic thermogenesis in mice (Zhang et al., 2019). Mechanistically, MC-derived serotonin inhibits the proliferation of PDGFRα^+^ adipocyte precursors that can be differentiated into beige adipocytes. An *in vitro* study also demonstrated that histamine degranulation in MCs triggers UCP1 expression in 3T3-L1 adipocytes (Finlin et al., 2019).

#### CD45^+^ hematopoietic cells

Interestingly, Jun et al. (2018) discovered that immune cells modulate thermogenic beige adipocytes through the peripheral cholinergic circuit in mice. During cold acclimation, acetylcholine (ACh)-secreting CD45^+^ hematopoietic cells (e.g. B cells, αβ T cells, and M2 macrophages) activated beige adipocytes via cholinergic receptor nicotinic alpha 2 subunit (Chrna2) (Jun et al., 2018). Taken in conjugation, these findings have reinforced the important role of adipose immune system in the regulation of beige adipocytes and energy metabolism.

## Endocrine factors for the formation of beige adipocytes

**Figure 4:**
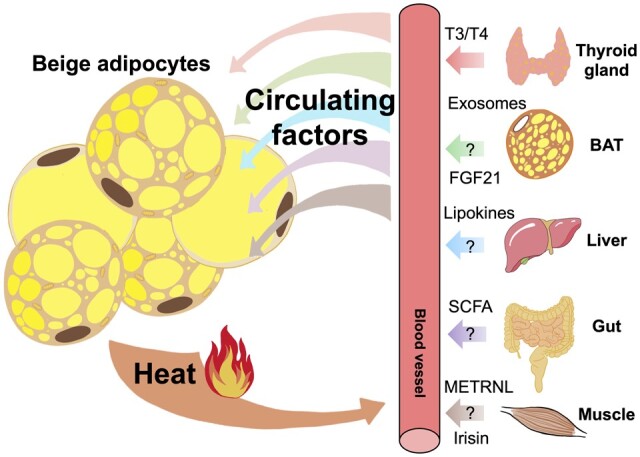
Circulating factors released from other metabolic organs modulate browning and thermogenesis of WAT through inter-organ communications. Question mark (?) represents other unknown factors and that the role of irisin in the browning of WAT is still controversial.

Apart from the local microenvironment, circulating factors can also act as endocrine signals to regulate the development of beige adipocytes in WAT (Figure 4).

### Peptide hormones

Upon cold exposure, shivering thermogenesis and non-shivering thermogenesis are involuntarily executed to counteract hypothermia. It has been suggested that cold-induced shivering stimulates the release of irisin into the circulation and also mimics exercise to induce the release of irisin during muscle contraction (Lee et al., 2014a). Irisin is known as an exercise-stimulated myokine that can induce the browning of WAT and improve parameters of metabolic complications in vivo (Boström et al., 2012). Nevertheless, the secretion of irisin from skeletal muscle is still controversial as some studies could not find the correlation between exercise and irisin level (Pekkala et al., 2013; Raschke et al., 2013). In addition, METRNL has been identified to be another PGC-1α-dependent myokine that drives the browning of WAT after exercise (Rao et al., 2014), suggesting crosstalk between muscle and fat in promoting the browning of WAT.

Besides myokines, the stress-responsive hormone FGF21 is selectively elevated in thermogenic adipose depots (BAT and subcutaneous WAT) during cold exposure and induces browning and adaptive thermogenesis of WAT (Fisher et al., 2012; Huang et al., 2017). FGF21 not only acts locally in an autocrine/paracrine manner but also exerts its actions as an endocrine signal to increase adaptive thermogenesis in WAT. BAT-secreted FGF21 can release into the bloodstream and travel to distal WAT to promote beige adipocyte formation (Hondares et al., 2011; Hanssen et al., 2015). However, cold exposure does not cause any significant change of circulating FGF21 (Fisher et al., 2012; Huang et al., 2017) or even leads to a decrease in the level of circulating FGF21 (Sepa-Kishi and Ceddia, 2018). It is possible that circulating FGF21 may be involved in beige adipocyte formation induced by other types of metabolic stresses but not by cold exposure. In addition, one of the most abundant adipokines, adiponectin, has been reported to be induced specifically in subcutaneous WAT but not BAT and epididymal WAT during cold exposure, which in turn activates the thermogenic program of beige adipocytes through the proliferation of M2 macrophages (Hui et al., 2015). However, why the circulating level of adiponectin is reduced during cold exposure remains obscure (Dong et al., 2013; Hui et al., 2015).

### Thyroid hormones

The thyroid gland releases two types of thyroid hormones, namely 3,3′,5,5′-tetraiodothyroxyne (T4) and 3,3′,5-triiodothyronine (T3). Thyroid hormones are key regulators of BAT-mediated adaptive thermogenesis in mammals. T4 is the major form of thyroid hormone in circulation and has to be converted to its activated form, T3, by type 2 iodothyronine deiodinase (Dio2). T3 can then bind to nuclear thyroid receptor alpha (TRα) or beta (TRβ) to exert its biological functions. A study has suggested that TRβ is involved in the regulation of adaptive thermogenesis through modulating UCP1 gene expression, while TRα appears to play a role in adrenergic sensitivity (Ribeiro et al., 2001). Accordingly, administration of GC-1, a TRβ-specific agonist, to *ob/ob* mouse model promoted significant browning of subcutaneous WAT, which led to an increase in body temperature and energy expenditure (Lin et al., 2015). This effect was also observed by chronic subcutaneous treatment with T4 or intracerebroventricular infusion of T3 in rodents (Martínez-Sánchez et al., 2017). However, this concept was challenged by another study reporting that systemic T3 induced hyperthermia in mice through actions in TRα-expressing skeletal muscle with central body temperature setpoint, but not the consequences of browning of WAT (Johann et al., 2019). Therefore, further studies are required to clarify the role of thyroid hormones in the browning of WAT in addition to its well-established central effect on BAT.

### Lipokines

It is increasingly appreciated that lipokines or bioactive lipids are important mediators for inter-organ communication (Cao et al., 2008). There is substantial evidence demonstrating the involvement of lipid breakdown in promoting brown and beige adipocyte-mediated adaptive thermogenesis, as this process utilizes fatty acids as fuels for heat generation (Lynes et al., 2018; Leiria et al., 2019). Metabolic organs such as AT (Cao et al., 2008; Yore et al., 2014) and the liver (Simcox et al., 2017) have been reported to release lipokines associated with improved metabolic health. For instance, through a global lipidomic analysis, a novel circulating lipid known as 12,13-dihydroxy-9Z-octadecenoic acid (12,13-diHOME) was found to increase in both mice and humans when exposed to cold or exercise, facilitating fatty acid uptake and thermogenesis in brown and beige adipocytes (Lynes et al., 2017; Stanford et al., 2018). Moreover, a cold-activated circulating mitochondrial phospholipid, cardiolipin, has been shown to bind to and increase the stability of UCP1 in the mitochondrial inner-membrane (Lee et al., 2015b; Lynes et al., 2018). Interestingly, AT macrophages have been shown to convert breast-milk alkylglycerol (AKG) into platelet-activating factor (PAF), which triggers and maintains beige adipocytes in infants. However, it remains unclear whether AKG promotes browning of WAT in adult humans and mice (Yu et al., 2019). A considerable effort has been made to identify lipokines involved in regulating the activity of classical BAT activation for thermogenesis, but further investigation is needed to clarify their roles in the recruitment and/or activation of beige adipocytes in WAT.

### Exosomes

Exosomes, also known as extracellular nanovesicles, contain microRNAs (miRNAs), non-coding RNAs, proteins, or bioactive lipids that are secreted by cells to mediate inter/intracellular communication for physiological and pathological conditions (Huang and Xu, 2021). In this regard, exosomes derived from adipose-derived stem cells (ADSCs) have been shown to promote the polarization of M2 macrophages, which in turn leads to WAT browning and subsequently improves metabolic disorders in dietary obese mice (Zhao et al., 2018). This finding was further supported by another study showing that exosomes isolated from human ADSCs enhanced the differentiation of beige adipocytes, suggesting that exosomes derived from beige adipocytes can stimulate the development of other beige cells in WAT (Jung et al., 2020). However, the exact exosomal components that mediate WAT browning and thermogenesis remain unknown. Notably, the level of a circulating exosomal miR-92a is found to be inversely correlated with brown/beige cells in humans and mice exposed to cold or mice treated with the β3-AR agonist CL316,243, suggesting that it can be used as a potential biomarker for brown/beige adipocytes in humans (Chen et al., 2016). Nevertheless, the role of miR-92a in thermoregulation has not yet been identified. Several previous studies have highlighted the role of endogenous miRNAs in WAT browning, including miR-155 and miR-196a (Mori et al., 2012; Chen et al., 2013). However, there is currently no evidence showing that these microRNAs exist in the circulating exosomes and act in an endocrine manner to modulate the browning of WAT. Therefore, further studies are needed to identify the precise exosomal components involved in the regulation of biogenesis of beige adipocytes.

### Gut microbiota-derived factors

Host‒microbiota interactions involve the production of metabolites that subsequently impacts metabolism and immune system function in the host. Recently, it has been reported that exposure to a cold environment altered the gut microbiota composition of mice. Transplantation of gut microbiota collected from mice exposed to a cold environment into germ-free (GF) mice increased the browning of WAT, cold tolerance, as well as energy expenditure (Chevalier et al., 2015). Furthermore, depletion of gut microbiota in mice by using GF and antibiotics treatment led to a browning phenotype in subcutaneous and epididymal WAT at 22°C and 30°C. They provided evidence showing that upon depleting the gut microbiota, the activation of type 2 immune response in WAT drives browning capacity, but the exact microbial-secreted factor(s) that mediate type 2 signaling pathway is currently unknown (Suárez-Zamorano et al., 2015). Furthermore, caloric restriction-induced gut microbiota remodeling leads to type 2 immunity-mediated browning of WAT via lipopolysaccharides‒Toll-like receptor 4 axis in mice (Fabbiano et al., 2018), which may explain the formation of beige adipocytes observed in the microbiota-depleted mice. In contrast, Li et al. (2019) demonstrated an opposite finding that the elimination of microbiota impaired the browning process of WAT and thermogenic capacity of BAT in mice. Recolonization of microbiota or replenishment with butyrate partially restored the impaired thermoregulatory response in microbiota-depleted mice, suggesting a potential role of microbiota-derived metabolites such as short-chain fatty acids (SCFAs, e.g. butyrate, acetate, and propionate) in thermogenesis (Li et al., 2019). Indeed, gut microbiota-derived acetate and lactate have been shown to promote the browning of WAT but not thermogenic BAT following intermittent fasting (Li et al., 2017). Further studies are necessary to evaluate and clarify the impact of gut microbiota composition in modulating the host thermogenic program of WAT.

## Challenges in the clinical translation

The discovery of brown/beige adipocytes in adult humans has triggered massive research interest because of its therapeutic potential to combat obesity and obesity-related abnormalities. However, most of the pre-clinical research outcomes have been heavily relying on the use of mouse models, which may not represent the exact phenomenon in humans. Although there are similarities between humans and mice, notable differences do exist. For instance, posterior subcutaneous WAT (from dorsolumbar to gluteal region), the most susceptible depot to beige fat biogenesis in mice is anatomically equivalent to the gluteal-femoral subcutaneous region in humans that does not have detectable brown/beige adipocytes (Chusyd et al., 2016; Leitner et al., 2017). Moreover, omental fat biopsy was found to express higher levels of browning and beige adipocyte gene markers than abdominal subcutaneous fat in humans, as compared to what was observed in mice (Zuriaga et al., 2017), suggesting that the depot-specific browning capacities of mice and humans are not comparable. Furthermore, information on the browning susceptibility of human WAT in different anatomical sites is still lacking.

Another critical difference between humans and mice is the thermoneutrality condition when studying the browning process. Unlike humans who live in a comfortable environment that is close to thermoneutrality, laboratory mice are constantly housed at ambient temperature (∼22°C), which is below their thermoneutral zone (∼30°C), consequently affecting many biological processes (Cannon and Nedergaard, 2011). It has been reported that mice housed at ∼22°C displayed energy expenditure (EE) 3.1-fold higher than the resting metabolic rate (RER), significantly exceeding EE/RER ratio in humans (∼1.6) (Fischer et al., 2018). Therefore, this chronic thermal stress in mice has contributed significantly to the outcomes of earlier metabolic studies (Feldmann et al., 2009; Overton, 2010), which may lead to unreliable results in subsequent metabolic change or overestimation of non-shivering thermogenic capacity in humans.

Activation of β3-AR and its downstream cAMP signaling cascade through chronic cold exposure or pharmacological intervention has been identified as potent thermogenic inducers of beige adipocytes in mouse models. However, the use of β3-AR agonists as anti-obesity medication in clinical trials has led to mixed outcomes (Arch, 2011). Pharmacological agents (e.g. repositioning of the β3-AR agonist mirabegron) have led to undesirable cardiovascular side effects in humans despite stimulation of brown fat and improved glucose homeostasis. It is also uncomfortable and time-consuming to expose individuals to a cold environment as a therapeutic approach. A recent study even showed that human brown/beige fat in the deep neck region is stimulated through β2-AR, but not β3-AR (Blondin et al., 2020). Finally, it has yet to be clarified whether the induction of the browning of WAT is sufficient enough to increase energy expenditure and decrease adiposity in humans (Schiffelers et al., 2000; van Baak et al., 2002). Therefore, results from animals should be extrapolated to humans with cautions.

## Concluding remarks and future perspectives

Despite enormous efforts, there are very few pharmacological interventions available to treat obesity and its related medical complications. Most of the anti-obese mediations, especially those acting centrally to suppress food intake, were withdrawn from the market due to their severe side effects after long-term administration. Given the existence of excess amounts of white fat in obese humans, browning of WAT represents a promising alternative strategy to combat obesity, as a high volume of thermogenic beige adipocytes can be readily achievable. Indeed, CRISPR-engineered brown-like adipocytes have been tested to treat obesity and its related metabolic syndromes (Tran et al., 2008; Stanford et al., 2013; Blumenfeld et al., 2018; Wang et al., 2020). However, this approach poses significant technology challenges in the manufacturing process. In addition, lack of cell‒cell communication between different cell types including nerves, endothelial cells, and immune cells may further influence the function and viability of the transplanted tissues/cells. Therefore, the identification of secreted factor/soluble molecules to induce/activate beige adipocytes to create a microenvironment favorable for biogenesis and thermogenesis of beige adipocytes may help to develop biopharmacological strategies to combat obesity by the browning of WAT. However, additional investigations are needed to explore novel delivery systems such as AT-targeted nanoparticles and lipid nanocarriers to specifically deliver the ‘browning’ molecules to WAT. This will provide not only an efficient and precise delivery of bioactive substances but also a marked reduction of side effects in other non-adipose organs.

Another important issue that needs to be addressed is to develop a high-sensitivity and high-resolution quantitative method for the measurement of brown/beige adipocyte mass/activities in humans. The current imaging technology such as PET/CT is too expensive and not suitable for routine assessment of brown/beige activity. In this connection, identification and characterization of circulating biomarkers specifically related to beige/brown adipocytes will be extremely useful for dynamic, non-invasive monitoring of brown/beige activities. To translate the findings from bench to clinic, further investigation is needed to dissect the similarities and differences between rodents and humans in the recruitment, activation, and maintenance of functional beige adipocytes.

## Funding

This work was supported by Hong Kong Research Grants Council/Area of Excellence (AoE/M/707-18), Collaborative Research Fund (C7037-17W), and General Research Fund (17125317).

**Conflict of interest**: none declared.
